# Supporting employers to enhance the return to work of cancer survivors: development of a web-based intervention (MiLES intervention)

**DOI:** 10.1007/s11764-019-00844-z

**Published:** 2020-01-14

**Authors:** M. A. Greidanus, A. G. E. M. de Boer, C. M. Tiedtke, M. H. W. Frings-Dresen, A. E. de Rijk, S. J. Tamminga

**Affiliations:** 1 Coronel Institute of Occupational Health, Amsterdam Public Health research institute, Amsterdam UMC, University of Amsterdam, Meibergdreef 9, Amsterdam, The Netherlands; 2 Department of Public Health and Primary Care, Centre for Environment & Health, Katholieke Universiteit Leuven, Kapucijnenvoer 35, Leuven, Belgium; 3 Department of Social Medicine, Faculty of Health, Medicine and Life Sciences, Research Institute Primary Care and Public Health (CAPHRI), Maastricht University, Duboisdomein 30, Maastricht, Netherlands

**Keywords:** Return to work, Intervention Mapping, Cancer, Internet-Based Intervention, Employer, Cancer Survivors

## Abstract

**Purpose:**

The purpose of this study was to develop an intervention targeting employers, with the aim of enhancing cancer survivors’ return to work (RTW).

**Methods:**

Intervention Mapping was used to combine information gathered from several procedures involving numerous stakeholders, for example, employers, cancer survivors, oncological occupational physicians, and e-health experts.

**Results:**

Employers indicated that they require tailored support during four RTW phases: (1) disclosure, (2) treatment, (3) RTW planning, and (4) actual RTW. The most important employer actions were identified for each RTW phase, for instance, “communicate,” “support practically,” and “assess work ability,” and thereafter formulated as the performance objectives of the intervention. The trans-theoretical model of change was used as a theoretical framework, and several methodologies were employed to induce the desired behavior change, for example modeling, tailoring, and active learning. Subsequently, a web-based intervention with interactive videos, conversation checklists, links to reliable external sources, and succinct, tailored tips and information was developed and adjusted on the basis of pre-tests with different stakeholders.

**Conclusions:**

The intervention was developed with input from employers and all relevant stakeholders in the RTW of cancer survivors. The systematic, step-wise development resulted in a succinct and easily accessible intervention targeting the most important employer actions during all RTW phases. As such, the intervention corresponds with employers’ needs and preferences in practice.

**Implications for cancer survivors:**

By providing employers with support, the intervention could well be the missing link in efforts to optimize the work participation of cancer survivors.

**Electronic supplementary material:**

The online version of this article (10.1007/s11764-019-00844-z) contains supplementary material, which is available to authorized users.

## Background

Since the survival rates of people with a diagnosis of cancer are increasing, appropriate cancer care entails more than just medical treatment [1]. A person’s quality of life during and after a treatment for cancer has therefore received more attention in recent decades [1]. An important aspect of the quality of life of cancer survivors is labor participation, as work can provide structure, a sense of normality, and social interaction [2–4].

Physical, psychological, and work-related barriers might prevent cancer survivors from remaining at work during treatment or from returning to work thereafter [5]. Multiple interventions have endeavored to tackle these barriers and thus facilitate the labor participation of cancer survivors [2, 6–8]. However, these interventions are primarily focused on the cancer survivor him- or herself, and although that appears to be an obvious choice, the effects of these interventions on the work participation of cancer survivors have unfortunately not been unambiguous [2, 6, 9].

From a broader perspective, support from the workplace was found to be a decisive factor in enhancing the labor participation of cancer survivors [10]. The key stakeholder in this broader perspective is the employer, as the employer is in a position to tackle work-related barriers by, for example, allowing a flexible return to work (RTW) plan and providing appropriate practical arrangements for the cancer survivor [11–15]. The role of the employer is therefore essential in facilitating the cancer survivor’s RTW.

From an employer’s point of view, facilitating a cancer survivor’s RTW is not as straightforward as one might think [16–18]. Employers perceive their role in this RTW as complex and demanding [17]. Several dilemmas are experienced by employers, for example, conflicting interests of the cancer survivor and the organization [17, 18]. Also, an employer’s lack of knowledge of cancer and inadequate communication skills, as well as inflexible national and organizational policies, might prevent the employer from giving appropriate RTW support to the cancer survivor [17, 18]. Several studies therefore recommend providing employers with guidelines and other supportive interventions to enhance the RTW of cancer survivors [2, 16–27]. As there was a lack of scientifically sound interventions targeting employers – which might well be the missing link in efforts to optimize the work participation of cancer survivors [16, 21] – we developed one, namely, the MiLES (“the Missing Link: optimizing the return to work of Employees diagnosed with cancer, by Supporting employers”) intervention.

A work-related intervention targeting employers is complex, considering, for example, the diversity among cancer survivors, the number of possible employer actions during the RTW trajectory of a cancer survivor, and the required degree of flexibility and tailoring concerning these actions [18, 22, 28]. In order to maximize the probability that an intervention will be effective, it is recommended to develop it systematically [29]. The purpose of the present research was to develop the MiLES intervention, which targets employers during the RTW of cancer survivors and is intended to enhance the cancer survivors’ RTW.

## Methods

The Intervention Mapping (IM) approach was used to structure the development of the MiLES intervention [30]. IM is one of the most comprehensive systematic approaches for the development of theory and evidence-based behavior change interventions, and it is a framework that is characterized by a participative approach involving the relevant stakeholders [29, 30]. This framework has been used regularly in the development and implementation of both cancer-related [31] and work-related interventions [32–35]. The IM framework consists of six consecutive steps, with the results of each step guiding the subsequent one: step 1, needs assessment; step 2, formulating objectives using a logic model of change; step 3, selecting theories and practical strategies; step 4, developing the intervention; step 5, planning for program adoption and implementation; and step 6, planning for evaluation [30]. Since this paper focuses on the development of the MiLES intervention, only steps 1–4 are presented here. Steps 5 and 6 of the IM framework will be taken in 2019 and 2020.

**Fig. 1:**
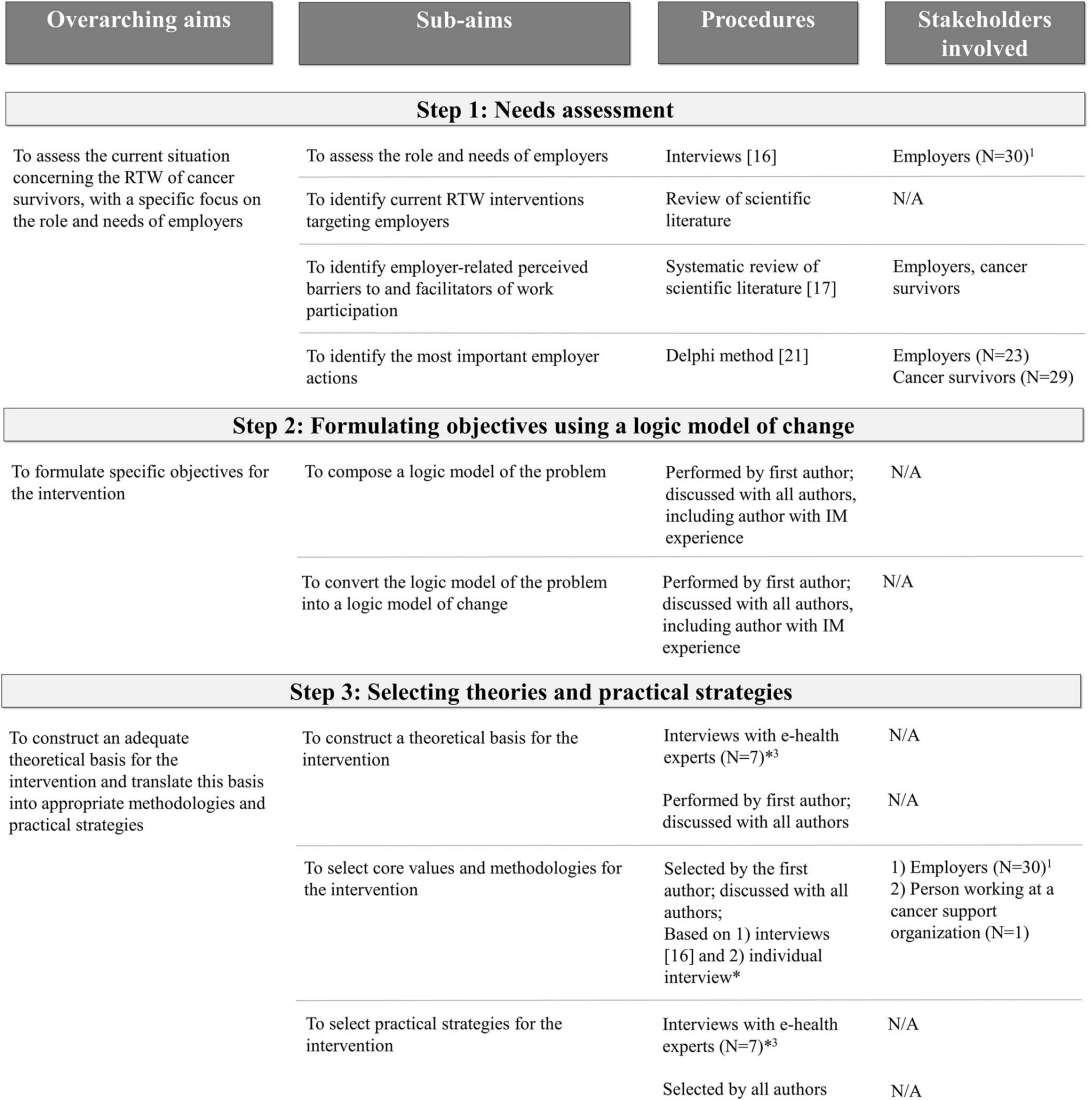

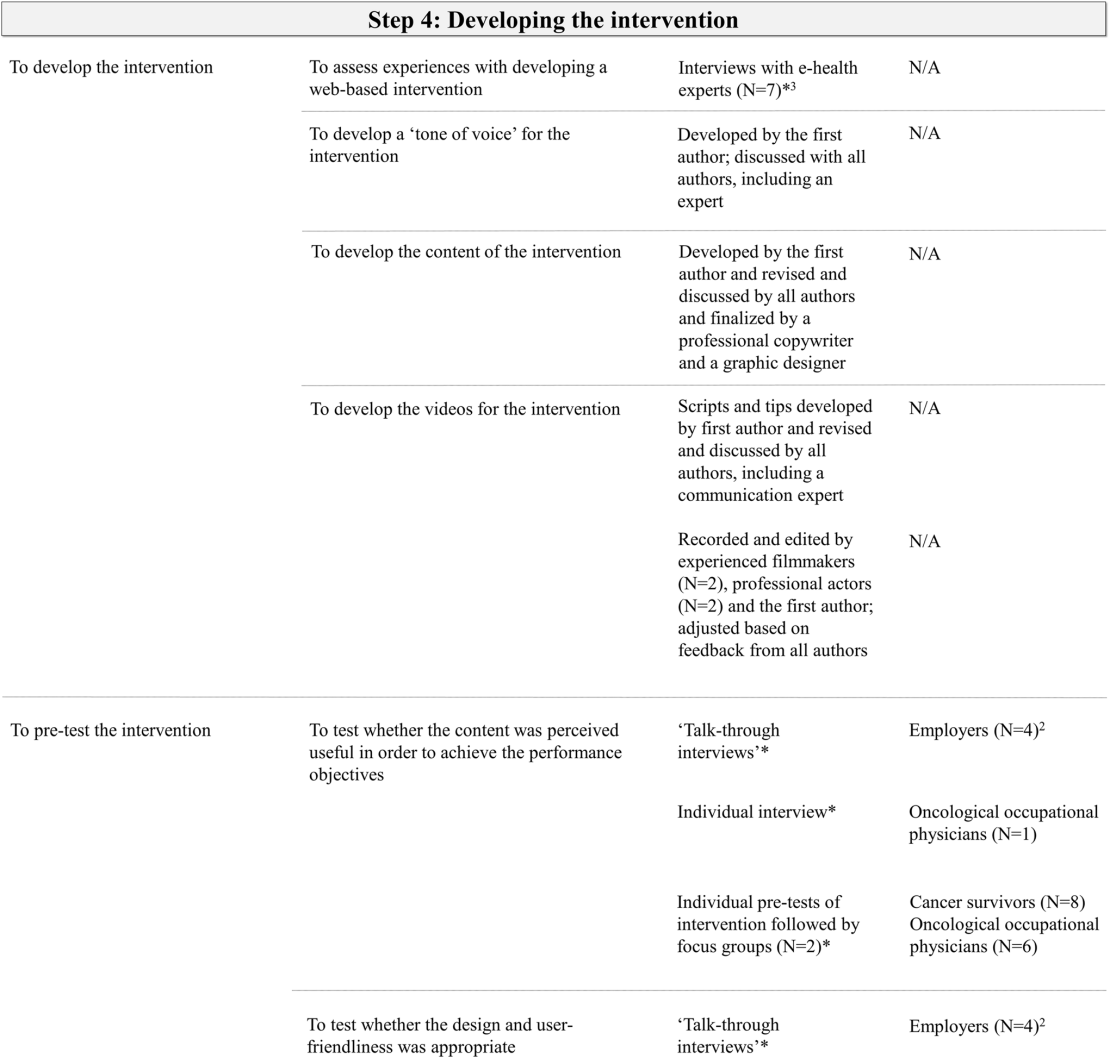
Overview of each IM step: overarching aim(s), sub-aims, procedures, and stakeholders in the RTW of cancer survivors involved in the development of the MiLES intervention. ^1^, ^2^, and ^3^: the same individuals were involved in these studies, respectively. N/A: not applicable. *: more information about this procedure can be found in Appendix A

For each IM step, one or two overarching aims and several sub-aims were formulated (Fig. 1). Various procedures involving numerous relevant stakeholders in the RTW of cancer survivors were used to obtain the desired information or to accomplish these sub-aims. The procedures included interviews, literature reviews, a Delphi study with expert panels, and meetings between the authors and with other relevant experts. More information about some of the procedures can be found in Appendix A.

## Results

### Step 1: needs assessment

#### Setting

In the Netherlands, employers have far-reaching legal responsibilities concerning RTW care for employees on sick leave, as laid down in the Dutch Gatekeeper Improvement Act, which requires the active involvement of employers in the work resumption program of employees, right from the start of an employee’s absence from work and continuing for a period of 2 years [36]. In addition to this active involvement, Dutch employers are obliged to continue 70% of the employee’s salary payment for 2 years of sick leave if needed, including job protection [36]. All Dutch employees also have, by law, access to an occupational physician, who provides them with health- and labor-related care when they are sick-listed [36]. Occupational physicians also advise employers concerning sickness absence procedures [36].

#### Needs assessment

The following concrete needs of employers regarding the RTW of cancer survivors were identified and used as input for subsequent IM steps:

Employers experience complex communication and decision-making during the RTW of cancer survivors [17]. The following RTW phases were identified:

1. *Disclosure*: the period between disclosure of the cancer survivor’s illness to the employer and the first treatment
2. *Treatment*: the period of sick leave during the cancer survivor’s treatment
3. *RTW planning*: the period in which the concrete planning and preparation of the cancer survivor’s RTW take place
4. *Actual RTW*: the period after RTW

Although the Dutch legislation on sick-listed employees is extensive, research revealed that this legislation does not support employers sufficiently in the case of cancer [17]. We therefore hypothesized that employers need to be supported in the active role they have during the RTW of cancer survivors. More specifically, employers need communication skills and information on how to support cancer survivors during the abovementioned RTW phases [17].

No scientifically sound interventions solely targeting employers to enhance the RTW of cancer survivors were identified [37]. Some patient-oriented RTW interventions involved the employer of a cancer survivor in the intervention by, for example, organizing an RTW meeting with the employer and making a gradual RTW plan in collaboration with the cancer survivor, employer, and occupational physician [38]. However, involving the employer in this type of RTW interventions turned out to be challenging [38]. It is therefore essential to assess the employers’ preferences regarding the type and design of the intervention (IM steps 3 and 4), in order to optimize the intervention engagement of employers.

A large variety of employers’ behaviors, attitudes, or perceptions were perceived as barriers to or facilitators of the RTW of cancer survivors, such as communication, knowledge about cancer, work environment, and perception of survivors’ work ability [18]. These barriers and facilitators were interpreted in terms of the employer’s willingness and ability to support a cancer survivor [18], in order to categorize different types of change objectives in IM step 2.

The abovementioned perceived barriers and facilitators were condensed into the most important employer actions, according to employers and cancer survivors [22]. The following employer actions were identified by cancer survivors and/or employers as most important for the successful RTW of cancer survivors: “emotional support,” “practical support,” “allow sufficient sick leave,” “plan return to work,” “adjust expectations,” “assess work ability,” “show appreciation,” “communicate,” “treat normally,” and “handle unpredictability.” Employers need support to perform these employer actions. The employer actions were all considered to be specific to one to three of the abovementioned RTW phases. It is therefore important that the intervention provides support that is specific to each of the RTW phases. Employers and cancer survivors mostly identified corresponding employer actions, but not during the survivor’s sick leave (RTW phase 2). Different perspectives might put effective collaboration between both parties at risk [22]. It is therefore of great importance to have a mutual understanding of each other’s needs and preferences throughout the RTW phases [22]. The perspectives held by the cancer survivors also differed considerably [22]. Employers should therefore provide cancer survivors with tailored RTW support.

### Step 2: formulating objectives using a logic model of change

A logic model of the problem was composed and subsequently converted into a logic model of change.

#### Logic model of the problem

For each of the most important employer actions (identified in IM step 1), a specific logic model of the problem was composed using the Resource Dependence Institutional Cooperation (RDIC) model of employer support [18], which is based on the RDIC model [39]. The RDIC model of employer support assumes that whether an employer performs a specific employer action properly is determined by the following two concepts:

1. *Willingness of the employer*, which may be influenced by the employer’s perception of the cancer survivor and the employer action
2. *Ability of the employer*, which may be influenced by the employer’s knowledge and skills, as well as by external factors (e.g., regulations or the organization)

The determination and distribution of possible underlying determinants were based on the various studies conducted for the needs assessment (IM step 1). As an example, the logical model for the employer action “emotional support” can be found in Appendix B.

#### Logic model of change

**Fig. 2:**
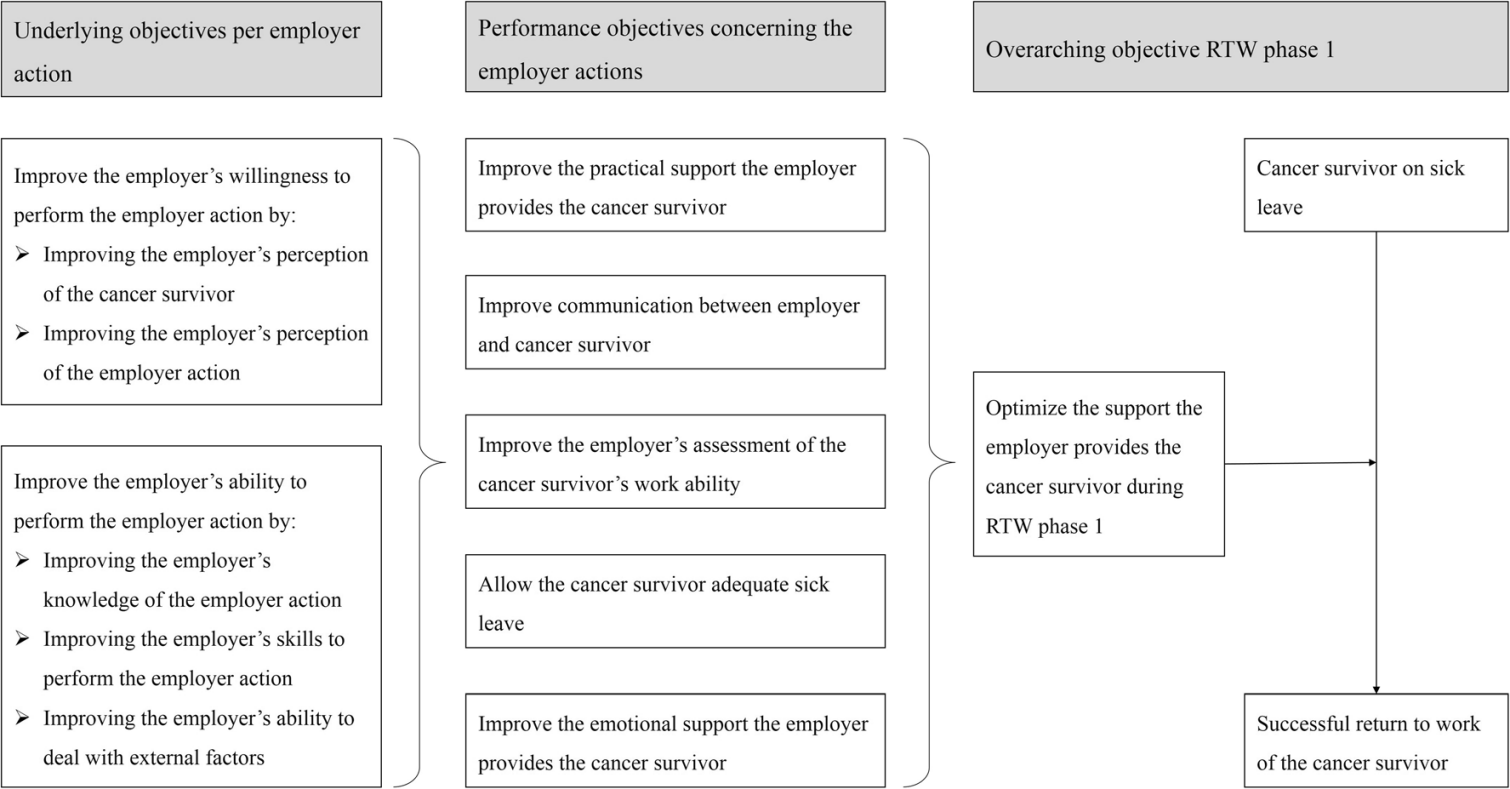
The various levels of objectives for RTW phase 1: the overarching objective, the performance objectives concerning the employer actions, and, per employer action, the underlying objectives related to the willingness and ability of the employer to perform the employer action. The RDIC model of employer support, which is based on the RDIC model [39], hypothesized that adequate employer support can enhance the successful RTW of cancer survivors on sick leave. The employer actions shown in the middle column are perceived as the most important employer actions for the successful RTW of a cancer survivor, according to survivors and employers participating in the Delphi study [22]

The objective of the intervention is to optimize the successful RTW of cancer survivors by supporting the employer. It was hypothesized that adequate support from the employer will optimize the successful RTW of a cancer survivor. In turn, it was assumed that support from the employer will be adequate if the employer is able to perform the most important employer actions throughout the different RTW phases and to tailor these based on the preferences and needs of his or her specific cancer survivor. As an example, the overarching objectives for RTW phase 1, including the performance objectives concerning the employer actions, and the underlying objectives per employer action related to both the willingness and the ability of the employer, are visualized in Fig. 2.

A specific logic model of change was composed for each employer action separately. While doing so, the logic model of the problem for that specific employer action was kept in mind. Detailed objectives were formulated to indicate the desired behavior. The performance objectives concerning the employer actions during each of the RTW phases, including specific objectives per employer action, all related to both the willingness and the ability of the employer, were the basis of the intervention (see Appendices C–F).

### Step 3: selecting theories and practical strategies

#### Theoretical basis

In order to induce the behavior change as formulated in the objectives of the intervention, the trans-theoretical model of change was used as a framework [40]. This model was chosen as it contributed to the understanding of behavior change, and structured the decision on which methodologies and practical strategies to use in order to induce the targeted behavior change. The following stages of behavior were distinguished for the intervention. Stage 1) pre-contemplation, the employer has no intention of executing the employer action; stage 2) contemplation, the employer intends to implement the employer action within a foreseeable time span; stage 3) preparation, the employer intends to implement the employer action directly; and stage 4) action, the employer has made specific adjustments to be able to implement the employer action (Table 1).

**Table 1:** Overview of the intended progress concerning the employer’s behavior change. The two columns on the right indicate the methodologies and practical strategies used to guide employers through the various stages of behavior change

Intended progress behavior change stages	Central question	Concept model of employer support	Methodologies	Practical strategies
Pre-contemplation ↓ Contemplation	Why is this employer action important?	Willingness of the employer	○ Information about personal risk ○ Scenario-based risk evaluation ○ Persuasive communication ○ Tailoring ○ Reinforcement	○ Succinct, tailored tips and information ○ Animation video
Contemplation ↓ Preparation	How should the employer action be implemented?	Ability of the employer	○ Active learning ○ Communication skills training ○ Persuasive communication ○ Tailoring ○ Modeling	○ Interactive videos ○ Succinct, tailored tips and information ○ Conversation checklists ○ Links to reliable external sources
Preparation ↓ Action	What is the next concrete action?	Employer action	○ Goal setting ○ Active learning ○ Tailoring ○ Reinforcement ○ Persuasive communication	○ Conversation checklists ○ Succinct, tailored tips and information

#### Core values and methodologies

The core values and methodologies of the intervention are (Table 1):

- *Practice oriented*: employers indicated that the intervention should align with their daily practice, including realistic and recognizable situations. We therefore included in the intervention *communication skills training* and *modeling* as methodologies [30], and offer the intervention at the moment that employers need it.
- *Succinct*: employers need to be provided with concise and adequate tips and information. Superfluous or repetitive information causes employers to lose interest and thereby leads to lower usage. We therefore chose to *tailor* the content of the intervention [30, 41].
- *Stimulating*: employers are not always keen to be involved in an RTW intervention for cancer survivors and to provide the required RTW support. We therefore aim to stimulate employers by using a *persuasive tone*, stating potential positive *reinforcement* as a result of behavior that the intervention aims to induce, pointing out hypothetical negative consequences if a certain employer action is not performed properly (*scenario-based risk evaluation* and *information about personal risk*), involving employers and interacting with them in the intervention (*active learning*), and letting employers set personal goals (*goal setting*) [30].
- *Trustworthy*: employers indicated that they need reliable information and support, in order to avoid unintentionally violating regulations (e.g., privacy regulations).

#### Practical strategies

Based on the core values and methodologies of the intervention, it was decided that the MiLES intervention should be a web-based intervention comprising interactive videos, conversation checklists, links to reliable external sources, and succinct, tailored tips and information (Table 1). The following strategies were chosen [30]:

- *Web-based intervention*: an online intervention provides employers with access to the intervention in the way they want it (e.g., via a mobile device, laptop, or tablet) and when they want it (e.g., just before an appointment with a cancer survivor). In order to make the web-based intervention easily accessible and to prevent unintended drop-out, a log-on should not be needed.
- *Succinct, tailored tips and information*: the content of the intervention should contain concise tips and information that is tailored at several levels: (1) per RTW phase, (2) per employer action, and (3) per “experience type” of cancer survivor [42].
- *Interactive videos*: communication should be addressed by interactive videos with recognizable conversations between employer and cancer survivor. These videos should visualize how to communicate in different situations and with different “experience types” of cancer survivors [42]. The web-based intervention should also contain an *animation video* about differences among cancer survivors, to make employers aware that they should tailor their RTW support to the individual needs of the survivor.
- *Conversation checklists*: the intervention should also contain downloadable conversation checklists that support employers during their conversations with the cancer survivor. These checklists should contain relevant topics and illustrative questions and provide space for the employer to take notes during the conversation.
- *Links to reliable external sources*: the intervention should substantiate its thorough scientific roots, detail the cooperation with a privacy expert, and refer to trustworthy external information sources and additional support services for more comprehensive information and support.

### Step 4: developing the intervention

#### The intervention: a web-based intervention

The MiLES intervention is an open-access, web-based intervention. On the homepage is a 1-min animation video about differences between cancer survivors, and a schematic representation of the four RTW phases. Employers are asked to indicate in which RTW phase his or her cancer survivor is currently in.

When the employer clicks on a certain RTW phase, the next webpage consists of content that is tailored to that RTW phase. This content includes an interactive video and the most important tips for that RTW phase. The interactive video starts with a brief scene with an employer and a cancer survivor, followed by interactive video frame footage in which the employer is asked which “experience type” corresponds with his or her survivor. For each of these experience types, a 1–3 min scene is shown, preceded by and concluded with tips on how to communicate with those who experience their diagnosis as such. Below this interactive communication video, the most important tips for that RTW phase are shown in a tab content widget. These tips are limited to five or six concrete, succinct tips that match the performance objectives concerning the employer actions for that RTW phase, as formulated in IM step 2. By clicking on a certain tip, more information is shown about why this tip is important and how to implement it. This information is in certain cases accompanied by an interactive video, a conversation checklist, or a link to external information sources or additional support services. These interactive videos visualize a specific situation including a scene with a good conversation and a scene showing common pitfalls. These scenes are preceded by and concluded with either the most important do’s or the most important don’ts for that specific conversation.

In addition to the content per RTW phase, the intervention consists of: 1) a webpage with additional information about how cancer survivors can experience their work situation during and after treatment; 2) a webpage with links to external information sources (e.g., about legislation, privacy regulations, and information about diagnoses and treatments) and additional support services (e.g., oncological occupational physicians and specialized reintegration services); and 3) a webpage with background information about the MiLES project and a substantiation of the thorough scientific roots of the intervention. Lastly, all content can be downloaded as PDF files and printed out.

#### Pre-testing

Four major adjustments have been made to the MiLES intervention as a result of pre-tests with employers, cancer survivors, and oncological occupational physicians. First, the number of conversation checklists has been increased. Second, a webpage has been added where all interactive videos and conversation checklists are collectively displayed. Third, the navigation of the intervention has been simplified, for example, by playing the interactive videos in an embedded light box, instead of in a new tab. Fourth, information about the possible long-term effects of the cancer survivor’s sickness and treatment has been added to the webpage of RTW phase 4. Some minor textual changes have also been implemented.

## Discussion

This paper described the development of the MiLES intervention, which targets employers during the RTW of cancer survivors. For this, the systematic IM approach was used to combine information gathered from several procedures involving numerous stakeholders in the RTW of cancer survivors. IM steps 1–4 have been completed, resulting in an open-access, web-based intervention consisting of interactive videos, conversation checklists, and succinct, tailored tips and information. By providing employers with support to fulfill their important though complex role in the RTW of cancer survivors, the MiLES intervention could well be the missing link in efforts to optimize the work participation of cancer survivors [16, 21].

Several tools and interventions for employers, both in the case of cancer and in the case of other chronic diseases, were identified in the gray literature [37, 43]. In scientific literature, developing and studying RTW interventions targeting employers is still in its infancy, and a broad range of challenges and opportunities have been formulated [16, 26, 44, 45]. In the specific area of the RTW of cancer survivors, the MiLES intervention is the first scientifically substantiated intervention solely targeting employers.

The MiLES intervention differs considerably from RTW interventions that target cancer survivors themselves, predominantly regarding the chosen practical strategies. For cancer survivors, multidisciplinary interventions consisting of, for example, counseling, biofeedback-assisted behavioral training, and an exercise program have been recommended [2]. Also an IM-based RTW intervention for breast cancer survivors consisted of an extensive hospital-based intervention by an occupational therapist, including several counseling sessions [46]. By contrast, the broad range of employers that were involved in the development of the MiLES intervention indicated that they are in need of succinct, easily accessible, online information. IM has been a helpful framework to ensure that the MiLES intervention will meet these specific needs of employers. As such, the MiLES intervention is expected to align their needs and preferences in daily practice, which will assumedly increase the employers’ use of and adherence to the intervention, and thereby the chance that the intervention induces the desired behavioral change.

There are also some similarities between the MiLES intervention and other interventions aimed at promoting the RTW of cancer survivors. For example, tailoring is an important aspect not only in the MiLES intervention but also in many other RTW interventions [7, 33, 46–48]. This is an effective technique for behavior change, since it personalizes information, provides less redundant information, and increases the intervention user’s attention [41, 47]. The variability in cancer survivors’ perspectives on work [42], their needs and preferences regarding RTW [22], and employers’ needs during the RTW of cancer survivors underline the importance of tailoring for the MiLES intervention [16, 17, 22, 42]. Most interventions tailor their content on the basis of a baseline questionnaire or an expert opinion [7, 33, 46–48]. The MiLES intervention, however, stimulates employers to tailor the content themselves by, for example, clicking on a certain RTW phase or the cancer survivor’s “experience type” in the interactive videos. This active learning challenges employers to actively think about their specific situation and the specific cancer survivor. In line with tailoring, communication also plays an important role in interventions aimed at promoting the RTW of cancer survivors [33, 46, 47, 49]. As effective communication is a prerequisite for a customized RTW plan, stimulating effective employer–cancer survivor communication is one of the main aims of the MiLES intervention.

A major part of the MiLES intervention is believed to be applicable and supportive for employers in other countries. That is because key elements of the MiLES intervention, e.g., communicating with a cancer survivor and tailoring the work-related support to the needs and preferences of the cancer survivor, are mentioned to also be essential actions for employers in other countries [14, 50–54]. Furthermore, a cross-country comparison of employer perspectives on supporting cancer survivors found that experiences and needs of employers are largely comparable [55]. Nevertheless, the RTW phases that structure the intervention might be depending on national legislation and in particular the required employer role. The national social security system in the Netherlands guarantees the continuation of 70% of an employee’s salary payment during 2 years of sick leave, if needed. Therefore, the RTW phase during treatment might often be filled in with (partial) sick leave, and consequently, the next phases have to focus on the planning of, and actual RTW. In countries with less income protection, cancer survivors might try to continue working during treatment and/or take unpaid leave [56]. This might require a less strict division between the guidance during treatment, the planning of, and the actual RTW. However, the content of the intervention will not differ that much, since monitoring is still needed and plans for necessary work adaptions have to be made. Also, the intervention is based on the extensive role required from Dutch employers during all phases of RTW, from the moment of disclosure until the RTW. An intervention targeting French employers would, for example, most likely not include the disclosure and treatment phase, since French employers are hardly involved in the guidance of cancer survivors during these phases [57]. We therefore recommend for further research, in each individual country, to study which elements of the MiLES intervention can be retained and which need adaption to the concerning system [27].

Further research on the MiLES intervention is being developed. As such, its perceived utility for employers and its effectiveness for the successful RTW of cancer survivors is being studied. We hypothesize that, because of the systematic development involving numerous stakeholders in the RTW of cancer survivors, the MiLES intervention will meet employers’ needs and be feasible in practice.

### Electronic supplementary material

ESM 1 (DOCX 17.5 kb)
