# Supplementary material for: Supporting employers to enhance the return to work of cancer survivors: development of a web-based intervention (MiLES intervention)
Source: J Cancer Surviv. 2020 Jan 14;14(2):200–10. doi: 10.1007/s11764-019-00844-z (PMC7182637; doi:10.1007/s11764-019-00844-z)
Supplement: Supplementary file 1 — (DOCX 17.5 kb) [file 11764_2019_844_MOESM1_ESM.docx]

***Appendix A: Comprehensive description of procedures used***

**Individual interview with person working at a cancer support organization**

IM step

Step 3: selecting theories and practical strategies.

Aim
To select the core values and methodologies for the intervention.

Participant
Person working at a cancer support organization (N=1). This person was designated by the concerning cancer support organization on the basis of experience and position.

Methods
The telephonic interview was performed by two authors (MG and ST), audio recorded and thereafter thematically processed by the first author (MG). On the basis of this processed interview, the first author formulated the main lessons learned regarding the MiLES intervention. These lessons learned were discussed and reformulated where appropriate during a meeting with all authors.

Topic list

The following topic list, developed by MG and checked by ST, structured the interview:

- What are the main support needs for employers participating in your organization’s program?
- How would you describe the differences between support needs of small and large sized organizations?
- How do you reach employers and encourage them to participate in your organization’s program?
- Can you elaborate on your organization’s choice for the methodologies and practical strategies used?
- What does the content of your organization’s program consist of?
- Are other stakeholders involved in your organization’s program?
- What components of your organization’s program are used most often?
- Can you describe how your organization individualizes the content of the program to different types of employers and cancer survivors?
- How do employers experience your organization’s program and components of this program?

**Interviews with e-Health experts**

IM steps

- Step 3: selecting theories and practical strategies.
- Step 4: developing the intervention.

Aims

- To construct a theoretical basis for the intervention.
- To select practical strategies for the intervention.
- To assess experiences with developing a web-based intervention.

Participants
Persons with experience in developing an e-health intervention (N=7).

Methods
All interviews were performed by the first author (MG), either telephonically (N=5) or face-to-face (N=2). Notes were written down during the interviews and thereafter processed and analyzed per topic. After all interviews, the most important lessons learned regarding the MiLES intervention were formulated by the first author and discusses and reformulated where appropriate during a meeting with all authors.

Topic list

The following topic list, developed by MG and checked by all authors, structured the interviews:

- What does your e-health intervention consist of?
- Why has the current content (website, app, etc.) been chosen?
- Have you used any behavior change theories or models in your e-health intervention, and how did you experience this?
- Have you integrated special strategies in your intervention to stimulate its used?
- Which website builder has built your intervention and what are your experiences with this website builder?
- How should the user log in to your intervention and what are your experiences with this?
- Do you keep log of the use of your intervention? What were your considerations in this and what are your experiences?
- How have you guaranteed the privacy of the users of your intervention? What strategies are implemented concerning the users’ privacy?
- How do you reach the users for the intervention?
- Which pitfalls do you have to take into account during the entire process of building, testing, using and implementation an e-health intervention?

**Talk through interviews with employers**

IM step

Step 4: developing the intervention.

Aims

- To test whether the content was perceived useful in order to achieve the performance objectives.
- To test whether the design and user-friendliness were appropriate.

Participants
Employers (i.e. direct supervisor, HR-manager or case-manager) with recent (<3 years ago) experience in guiding a cancer survivor at work (N=4). Employers were recruited via social media and via personal contacts of the project team. The included employers worked for non-profit (education, N=2) and profit (transport and financial sector, N=2) organizations.

Methods
All face-to-face, individual interviews were performed by the first author (MG). Each interview consisted of four parts and lasted 60-90 minutes in total. The first part consisted of some questions about the participant’s expectations of the intervention. During part two, participants were sitting at their personal computer or laptop and the interviewer outlined a specific situation as an introduction, for example: ‘Your employee has been diagnosed with cancer and is fully sick listed for two months now. You will visit the intervention looking for information and tips on how to support this cancer survivor in this specific situation. How would you navigate on the intervention and what do you think about the design and content of the intervention?’ After this introduction, the participation were encouraged to think out loud while he or she tested the web-based intervention. During this situation specific test, the interviewer asked specific questions to clarify the participant’s thoughts and opinions about the content, design and user-friendliness of the intervention. During the third part participants were asked to state some strengths and weaknesses of the intervention. The last part consisted of some specific questions concerning topics that have not been discussed during the previous parts of the interview, for example about the navigation, design, language used and content of the intervention.

All interviews were audio recorded and the first author extracted all suggestions from these audio records. The suggestions were assessed during a meeting with the authors and were implemented when they were: 1) in concordance with the performance objectives of the intervention, 2) feasible within the budget and timespan of the project, and 3) allowed within the legislations and privacy regulations.

Topic list

The following topic list, developed by MG and checked by all authors, structured the interviews:

Part 1:

- What do you expect from the intervention?
- What information do you expect to find on the web-based intervention?

Part 4:

- Do you understand the intervention’s navigation? Do you have any suggestions?
- When you click on a video, a new tab opens. Do you find this useful or inconvenient?
- What do you think about the RTW phases on the homepage? Is this clear enough or do you have any suggestions?
- How do you experience the design of the intervention?
- What do you think about the pictures on the intervention?
- What do you think about the use of language and the legibility of the content?
- What do you think about the printable conversation checklists? Would you use them?
- What do you think about the videos? Would you watch them?
- Are the videos sufficiently publicized on the intervention or would you, for example, place them directly at the top of every webpage?
- What do you think about the duration of the videos?
- What do you think about the interactive elements in the videos?
- Do you have any suggestions for the videos?
- Are you missing features or information on the intervention?
- When you think out of the box: what else would be helpful for you as an employer in order to support you during the RTW of a cancer survivor?

**Individual interview oncological occupational physician**

IM step

Step 4: developing the intervention.

Aim

To test whether the content was perceived useful in order to achieve the performance objectives.

Participant
An oncological occupational physicians (N=1). This physician was recruited via an email to all registered oncological occupational physicians and selected for an individual interview on the basis of experience in the field of cancer and work.

Methods
This face-to-face, audio recorded, individual interview of 60 min was performed by the first author (MG). The participant was asked to test the intervention and provide feedback on its content. The first author extracted all suggestions from this audio record and these suggestions were assessed during a meeting with the authors. The suggestions were implemented when they were: 1) in concordance with the performance objectives of the intervention, 2) feasible within the budget and timespan of the project, and 3) allowed within the legislations and privacy regulations.

Topic list

The following topic list, developed by MG and checked by all authors, structured the interview:

- What are the strengths and weaknesses of the intervention? Can you clarify?
- Do you have any additions for the intervention, for example, useful links?
- Should certain information on the intervention be removed, for example because this does not benefit cancer survivors’ situation?
- How can we encourage employers to use the intervention?

**Individual pre-tests by cancer survivors and oncological occupational physicians, followed by focus groups**

IM step

Step 4: developing the intervention.

Aim

To test whether the content was perceived useful in order to achieve the performance objectives.

Participants

- Cancer survivors diagnosed with cancer <5 years ago, >18 years old at time of diagnosis and currently working for an employer (N=6). Cancer survivors were recruited via social media.
- Occupational physicians with a completed specialization in cancer (‘oncological occupational physicians’) (N=8). Physicians were recruited via an email to all registered oncological occupational physicians.

Methods
Two separate focus groups were organized, one with cancer survivors and one with oncological occupational physicians. The focus groups were moderated by two authors (MG and ST) and were audio recorded. The focus groups started with a presentation of the objectives of the focus group. Thereafter, participants were asked to test the intervention individually on their own laptop or tablet for a period of 20-30 minutes. For this, every participant was asked to test a specific part of the intervention, for example RTW phases one and two. Participants were asked to fill out a pre-designed entry form with strengths and weaknesses of the intervention, and other experiences while testing the intervention.

Directly after the individual tests, the participants’ experiences were discussed with the other participants, during a meeting of 30-45 minutes. The moderator facilitated this group conversation to make sure all participants were able to share their opinion and thoughts.

The first author (MG) extracted all suggestions from the audio tapes and checked whether additional suggestions were given on the entry forms. The suggestions were assessed during a meeting with the authors and were implemented when they were: 1) in concordance with the performance objectives of the intervention, 2) feasible within the budget and timespan of the project, and 3) allowed within the legislations and privacy regulations.

Topic list

The following topic list, developed by MG and checked by all authors, structured the focus groups:

- What are the strengths and weaknesses of the intervention? Can you clarify?
- Do you have any additions for the intervention, for example, useful links?
- Should certain information on the intervention be removed, for example because this does not benefit cancer survivors’ situation?
- When you think out of the box: what else would be helpful for an employer in order to support them during the RTW of a cancer survivor?
- How can we encourage employers to use the intervention?
